# Broad concepts and messy realities: optimising the application of mental capacity criteria

**DOI:** 10.1136/medethics-2021-107571

**Published:** 2021-08-02

**Authors:** Scott Y H Kim, Nuala B Kane, Alexander Ruck Keene, Gareth S Owen

**Affiliations:** 1 Department of Bioethics, Clinical Center, National Institutes of Health, Bethesda, Maryland, USA; 2 Department of Psychological Medicine, IoPPN, London, UK; 3 Dickson Poon School of Law, King's College London, London, UK; 4 39 Essex Chambers, London, UK

**Keywords:** decision-making, competence/incompetence

## Abstract

Most jurisdictions require that a mental capacity assessment be conducted using a functional model whose definition includes several abilities. In England and Wales and in increasing number of countries, the law requires a person be able to understand, to retain, to use or weigh relevant information and to communicate one’s decision. But interpreting and applying broad and vague criteria, such as the ability ‘to use or weigh’ to a diverse range of presentations is challenging. By examining actual court judgements of capacity, we previously developed a descriptive typology of justifications (rationales) used in the application of the Mental Capacity Act (MCA) criteria. We here critically optimise this typology by showing how clear definitions—and thus boundaries—between the criteria can be achieved if the ‘understanding’ criterion is used narrowly and the multiple rationales that fall under the ability to ‘use or weigh’ are specifically enumerated in practice. Such a typology-aided practice, in theory, could make functional capacity assessments more transparent, accountable, reliable and valid. It may also help to create targeted supports for decision making by the vulnerable. We also discuss how the typology could evolve legally and scientifically, and how it lays the groundwork for clinical research on the abilities enumerated by the MCA.

Most jurisdictions require that a mental capacity assessment be conducted using a functional model whose definition includes several abilities. In England and Wales, the Mental Capacity Act (MCA) requires a person be able to understand, to retain, to use or weigh relevant information and to communicate one’s decision.^1^ Variations of these criteria are a part of laws in Australia, New Zealand, Singapore, Ireland and Northern Ireland.^2–7^

Although a functional model is an advance over the outdated and easily abused diagnostic (‘senility,’ ‘unsound mind’) criteria for incapacity,^8^ interpreting and applying broad and vague criteria such as ‘to use or weigh’ to a diverse range of clinical presentations will remain a challenge. Unfortunately, neither the MCA Code of Practice^9^ nor the recent National Institute for Health and Care Excellence guidelines on decision making and mental capacity^10^ provide detail on the conceptual contours of these criteria or their relation to clinical phenomena. This is not an optimal state of affairs for a practice that is quite challenging for evaluators to implement,^11^ where unstructured assessments can show poor reliability,^12^ and that some have even advocated we abandon wholesale.^13^

One obvious source of concrete guidance is the Court of Protection^i^ in England and Wales, a specialist mental capacity court created by the MCA whose judges apply the codified functional test, often with specialist clinical (usually psychiatric) evidence.^14–16^ A small (but large in absolute numbers) subset of court cases, either because of their subject matter (eg, serious medical treatment) or the level of judge to which they are allocated, are published based on guidance established by a senior judge.^17^

In an effort to help close the ‘translational gap’ between broad capacity criteria to more concrete guidance in applying them, we previously analysed 131 published court judgements on capacity regarding a wide range of decisions (about medical treatment, residence and/or several other issues) involving persons with dementia, intellectual disability, psychoses and other mental disorders.^18^ The analysis resulted in a typology of nine rationales that underlie the courts’ determinations of capacity. That analysis was ‘descriptively normative’—that is, it described and summarised the practices of a de facto source of normative authority, namely, the courts.

In this article, we move to a more critically normative analysis of the courts’ practices in applying the MCA criteria.^ii^ We attempt to optimise the de facto normativity of the judges’ views by critiquing the strengths and weaknesses of their practices, as well as to provide a conceptually coherent way to employ the typology of capacity rationales derived from their practices. We do not assume that the typology is exhaustive or final, only that such typologies can be useful insofar as they are clinically grounded and legally defensible.iii

We first introduce the descriptive typology of rationales used by the courts and point out some shortcomings. We then optimise that typology by applying a critical lens to the judges’ practices as well as to some of the ambiguities in the MCA criteria. This will naturally lead to concrete recommendations for applying the MCA criteria. We then address potential objections to the recommendations.

## A descriptive typology of judges’ rationales for their capacity determinations

We analysed 1416 textual units from 131 written court judgements and coded the justifications (rationales) that judges and expert witnesses used in their capacity determinations over a 10-year period.^18^ Using qualitative content analysis, we summarised the variety of rationales into a typology consisting of nine categories (two of which have three subcategories), as shown in the table 1.

**Table 1:** Typology of capacity rationales from 131 Court of protection and Court of appeal cases, 2008–2018^18^

Rationale	Description	Inability example quote	Intact ability example quote	MCA criteria most often linked to each rationale*
1. To grasp information or concepts	Unable to grasp, on a purely intellectual level, concepts (their nature or meaning) or information (eg, volume, detail, complexity) relevant to the decision.	[P] has barely an inkling of the health risks involved. She was unable to link sex to pregnancy. Indeed she had virtually no idea how her babies came to be in her tummy (as she put it)	He understands that the relationship is exclusive, and in broad terms that marriage includes society, support and assistance, and the concept of sharing a common home and domestic life, and that two people come together and owe each other rights and responsibilities	**Understand** (90%); Use or weigh (12%)
2. To imagine or abstract	Unable to imagine or abstract and therefore has difficulty considering relevant factors, including options, which are not concretely present or familiar.	She struggles with abstract thought such as picturing herself in a different setting	He understands that there is a choice between home or an institution and living with his family and he prefers the latter	**Use or weigh** (55%); Understand (45%)
3. To remember	Unable to remember facts or events that are needed to make the decision.	He had no memory of making the two LPAs	It was also clear to me that he had retained information given to him at various stages about these matters, including information imparted during the sex education sessions he has attended.	Understand (59%); **Retain** (45%)
4A. To appreciate: delusions/confabulations	Unable to apply information (including consequences of the decision) to oneself due to delusions or confabulations.	[P] believes that the tumour was placed in her body by ‘screen things’ with the aim of influencing the doctors into stating that the operation was needed	The view that [P] wishes to put forward is that she does not want the case to continue and she would prefer to stay where she is… I do not think her view is unreasonable or driven by delusion.	Understanding (60%); **Use or weigh** (51%)
4B. To appreciate: insight into condition or care needs	Unable to apply information (including consequences of the decision) to oneself due to lack of insight into one’s condition or associated care needs.	[P] denies that she suffers from schizophrenia, that she needs to take medication to remain well and avoid consequent relapse of her illness and renal failure. As a result she does not understand the need for supported accommodation.	She demonstrated an understanding of and insight into her care needs and the reality of life if she returned home. She clearly understands that she is in need of total support and would need carers to visit four times a day. Although she said she could dress herself ‘if I had to’, I did not interpret this as indicating a significantly exaggerated or distorted view of her capabilities. On the contrary, I found her to be broadly realistic as to her physical limitations.	
4C. To appreciate: other	Unable to generally apply information (including consequences of the decision) to oneself.	The point is that despite the overwhelming evidence to the contrary, [P] does not begin to appreciate that [Q] will not, under any circumstances, look after him	She denied inappropriate use of social media (‘I have kept away from social media … I don’t want to go back to square one’), showing an understanding that people contacting her through social media ‘might be a risk to me’	
5. To value or care	Unable to care about or value issues relevant to the decision hence unable to seriously consider certain options. This could relate to generalised apathy, or a strong attachment, fear or other emotion which overwhelms ability to value relevant information.	The compulsion to prevent calories entering her system has become the card that trumps all others	[P] [is] acknowledging her prognosis and choosing to give it no weight as against other information within the context of her own values and outlook when making a decision	**Use or weigh** (91%); Understand (13%)
6. To think through the decision non-impulsively	Unable to think through the decision and proceeds to make the decision impulsively or to act in impulsive manner.	The frontal lobe damage…means that a person such as [P] works on impulse. If the frontal lobe is disengaged from the decision-making process the decision is not thought out	None.	**Use or weigh** (88%); Understand (19%)
7A. To reason: flexible thinking	Unable to carry out basic mechanics of reasoning, specifically to employ flexibility of thought in responding to contrary evidence or concerns.	If [P] developed a fixed idea about a subject, it was very difficult for her to incorporate counterbalancing or conflicting information	[It is] not the case that [P]has undertaken the decision making exercise in relation to dialysis solely on the basis of a concrete or ‘black and white’ view taken in respect of her prognosis but rather on the basis of placing in the balance many factors relevant to the decision	**Use or weigh** (87%); Understand (14%)
7B. To reason: balancing pros and cons	Unable to carry out basic mechanics of reasoning, specifically to compare pros/cons, advantages/disadvantages or benefits/risks of the decision.	She cannot at the moment weigh the evidence up, identifying the pros and cons of a particular course of treatment, or really think about it at all. He said that when confronted with the balancing exercise she simply becomes both distressed and disengaged.	[P] gave [Dr X] a clear indication that she could weigh up the positives and negatives of whether or not to engage in sexual behaviour	
7C. To reason: other	Unable to generally carry out basic mechanics of reasoning.	She acknowledged receiving letters from [Q]. But she became significantly distressed, thought-disordered and preoccupied when invited to consider whether she might wish to respond to those letters	After consideration, he suggested two solutions which may not be implementable but are reasonable alternatives to consider. In so doing, he demonstrates an ability to think systematically and problem solve.	
8. To give coherent reasons	Unable to give any reasons for their choice or only able to give reasons which are internally contradictory.	He was not able to give coherent reasons for wishing to live where he is	She is nevertheless able to describe, and genuinely holds, a range of rational reasons for her decision. When I say rational, I do not necessarily say they are good reasons, nor do I indicate whether I agree with her decision	**Use or weigh** (63%); Understand (37%)
9. To express a stable or consistent preference	Expresses different or contradictory preferences at different times such that it is difficult to ascertain or to carry out the choice.	[P]’s more recent views about sterilisation have [not] shown any greater reliability, oscillating between being vehemently opposed to it, to requesting it immediately (and being distressed when this could not be arranged), before reverting to opposition.	[P] understands her preferences clearly and has maintained her position consistently over the three conversations she has had with him, namely that she is prepared to continue to live where she is now.	**Communicate** (44%); Use or weigh (44%)

*Only the two most often linked MCA criteria are listed as they account for the vast majority of linkages. Percent values can add to greater than 100% because court judgements sometimes give rationales with links to more than one MCA criterion.

MCA, Mental Capacity Act 2005.

How are these nine rationales related to the MCA criteria? To determine this, for each mention of a rationale, we coded the MCA criterion that was cited in that context. In theory, this should have allowed us to create a list of rationales that fell under each MCA criterion. For example, this method should have allowed us to mark the conceptual boundary between ‘understand’ and ‘use or weigh,’ with each criterion having its own list of distinct rationales used in applying that criterion. The results, however, were not that simple.

The column on the far right in table 1 shows, for each rationale type, the two most frequently linked MCA criteria in the judgements which account for vast majority of the linkages for each rationale. For example, the first rationale (to grasp information or concepts) was most frequently linked to the MCA ability to understand (90% of the time) but also sometimes to the ability to use or weigh (12% of the time; they can add to greater than 100% because the court judgements sometimes cite more than one MCA criterion in association with a given rationale.)

As can be seen, although for some rationales there is one overwhelmingly dominant MCA criterion that is linked to them (eg, to grasp information or concepts, to value or care, etc), for others that is not the case (eg, to appreciate). Furthermore, the MCA criterion of understanding appears as one of top two linked criteria for eight of nine rationales while the use or weigh criterion appears for six of the nine rationales. It, therefore, appears that (1) the MCA criteria (especially the understanding and use or weigh criteria) are used very broadly to cover many rationales and (2) the MCA criteria overlap considerably in use.

This does not seem ideal. That some criteria may have broad meanings is not in itself a problem since it may be that decision making as a concept involves complex abilities—a point that may be particularly pertinent to the criterion of use or weigh. However, when one MCA criterion—understanding—is one of top two criteria linked to virtually every type of rationale (eight of nine), one must wonder how practically useful it is to say that a person fails to meet the understanding criterion if it can, in effect, mean nearly any kind of impairment in decisional ability. And when this indiscriminate use contributes to overlapping boundaries of the MCA criteria, the potential for less than ideal practice of capacity evaluations is increased. For example, one evaluator can provide a rationale to justify an opinion that P is incapacitated because P fails to understand whereas another evaluator can appeal to the very same rationale (ie, the very same phenomenon) to argue that P lacks capacity because P fails on the use or weigh criterion. This creates potential for confusion and messy communication that could undermine the transparency and accountability in capacity assessments. Not only would such a situation make the teaching of (and providing guidance for) capacity evaluations rather complicated, it could also erode trust in the law as an objective and reliable means of protecting the self-determination rights of individuals. Such overlapping criteria would also make empirical research on mental capacity difficult to operationalise.

We suspect that most persons charged with evaluating capacity expect that the criteria which make up MCA’s conception of decision-making capacity are distinct without overlap. This view would be warranted since it would be odd if the legislators intentionally created an overlapping list of criteria without explaining why such a system was necessary. It would run contrary to a basic proposition of statutory drafting that each individual term should be doing distinct work. As we explain in the final section below, we believe that the legislative history of the MCA supports the presumption that the criteria are not intended to overlap. At any rate, regardless of intentions, it does not seem an ideal situation.

## Optimising the typology

Can the situation be remedied? Our ‘critically normative’ approach addresses this question as follows. We begin with the courts’ practice—as reflected in the typology of rationales—and critically interpret that typology so that each MCA criterion has as distinct a boundary as possible, minimising the overlaps between MCA criteria. In order to do this, we need to first understand why the court judgements contain multiple MCA criteria linked to a given rationale.

One reason why there seems to be an overlap in MCA criteria for some rationales is that, in many instances, the overlap is more apparent than real. This reflects suboptimal practices and/or artefacts of our method of qualitative analysis. The courts are not always meticulous in how they talk about a rationale in relation to the MCA criteria. Indeed, the most common manifestation of this is when judges provide rationales to justify their decisions without naming (and therefore do not explicitly link) the MCA criteria that they are presumably applying; this happens in 42% of the times that they give rationales for capacity judgements.

When judges do state the MCA criteria with their rationales, they often recite two or more MCA criteria in the discussion of a rationale but do so in ways that make the linkage more an artefact than a genuine conceptual link. For example, a judge might mention two MCA criteria together as a turn of phrase (eg, ‘understand and retain’) so that some MCA criteria are ‘swept up’ in the coding. In other instances, misleading links between rationales and MCA criteria are made because the judgements sometimes do not make explicit the hierarchical relationships between the criteria.^18^ For example, if P fails to comprehend some information (rationale 1), P cannot *use or weigh* it either, but the latter failure is secondary to the fact that P fails to understand. To state in such cases that P fails to use or weigh the relevant information in association with rationale 1 is true but superfluous. A similar point can be made about rationale 8 in table 1 (ability to give coherent reasons) where reason-giving is a downstream marker, that is, without being able to understand one would not be able to give coherent reasons.^18^ Here, the link with the understanding criterion is superfluous since the question of whether a person can give a coherent reason for a choice does not even become a live question if the person cannot absorb basic relevant information in the first place.

The point about these artifactual linkages to multiple MCA criteria is that they are only apparent linkages and therefore provide reasons for reducing some of the linkages. Some or most linkages to the criterion of understanding for rationales three and five through eight can be attributed to this phenomenon.

The second main reason why some rationales have more than one MCA criterion linked to them is that, for some rationales, the MCA criteria have ordinary English meanings that actually *can* overlap because they can be used in multiple senses. The challenge is therefore to provide clear practice guidelines that restrict the scope of some criteria so that they are consistent with the law (especially not creating any gaps between the criteria) while providing distinct boundaries that will be less prone to create confusion.

This can be accomplished mainly by recognising that the understanding criterion is especially prone to ambiguous uses, and by limiting it to a single use that has clear boundaries. Currently, neither the text of the MCA nor any published guidance documents explicitly restrict the meaning of ‘ability to understand’ to just one specific use, leaving ample opportunities for confusion. But we can distinguish between a specific narrow versus various other broader uses of the understanding criterion. The narrow use can be understood as the ability to comprehend intellectually—grasping of information and concepts—where it refers to the ability to absorb information.^iv^ Call this ‘understanding-1’. In essence, understanding-1 and the rationale of ‘grasping of information and concepts’ are the same. In our view, the understanding criterion should be used—when the term is invoked as an MCA concept—only in this sense.

Other uses of the term ‘understanding’ function to cover various things that one does with, or that happens to, the information so absorbed. To the extent that these can be accounted for by other MCA criteria, there is no loss or gap created by limiting the understanding criterion to understanding-1. We can put these other uses under ‘understanding-2’ which can come in various forms.^v^ Imagine one is talking with a person with significant short term memory impairment. The person can at the moment grasp the information (eg, can repeat back the information in own words) but fail to remember the information even after a minute or two. It is natural to say, ‘Oh, I’m afraid he doesn’t understand [understand-2].’ This can explain why ‘to remember’ (rationale 3 in table 1) is linked to both the retain criterion and the understanding criterion. In such situations, the capacity evaluator should simply appeal to the retain criterion.

Consider also the rationale of ‘to appreciate’ (applying the relevant information to one’s own situation).^vi^ We often use ‘understanding’ to mean appreciation: ‘Yes, he says he understands [understanding-1] what the treatment is for, but do you think he truly understands [understanding-2] how it will affect his life if he doesn’t receive it?” In this context, ‘understanding-2’ means being able to apply the information to oneself. But since ‘to appreciate’ can be accounted for by the use or weigh criterion, there is no gap created in limiting the use of appreciation rationale to the use or weigh criterion.

Consider next rationale 2 (ability to imagine or abstract). If a person is unable ‘to imagine or abstract and therefore has difficulty considering relevant factors, including options, which are not concretely present or familiar,’^18^ it is not unnatural to describe such a person as failing to understand. But if the person at some level comprehends (understanding-1) an idea (eg, that she may need to live at a new location) yet is unable to imagine what it would be like to live in that new location, it is just as (or more) natural to say this person is failing to *use* the relevant information. Thus, we recommend that rationale 2 be restricted to justifying the use or weigh criterion.vii

In summary, with the exception of rationale 9 (see below), our analysis does not need a customised justification of each rationale-MCA criterion linkage. Our critically normative analysis (1) begins with a normatively defensible starting point of the typology in table 1 and then (2) applies general critical considerations (suboptimal or ‘loose’ uses of MCA criteria as well as the recognition that the concept of understanding has specific narrow and various broad senses) to arrive at an optimised typology, one in which each rationale has a single MCA criterion linked to it (in table 1 with the preferred MCA criterion in bold).

We can summarise the results as follows. Rationale 1 should be linked only to understanding since it is the dominant link in practice and the link to use or weigh tends to be artifactual in this context. Furthermore, there is a general benefit of limiting understanding to this first rationale that extends to other rationales as well, as it leads to other MCA criteria having clearer and distinct boundaries. Thus, rationales 2 and 4 become limited to use or weigh criterion since that is the remaining link once understanding is restricted to rationale 1. The same applies to rationales 3, 5, 6, 7 and 8; these rationales, as noted above, also overlap with understanding partly as artefacts, further supporting this move to linking a single MCA criterion for each rationale.

Finally, the case of rationale 9 is somewhat unique. When a person is unable to provide a consistent or stable preference, it seems reasonable to interpret this as a failure to communicate a choice (contra the criterion’s legislative historyviii) since the assessor cannot determine what the ‘real’ choice or decision is. No speculation is involved here; the assessor simply does not have access to the person’s real choice. However, to attribute to such a person an inability to use or weigh is an inference about some presumed underlying impaired ability. In such cases, it seems better to justify the judgement with the MCA criterion of communication since if further probing (which should always be sought) ends up revealing another impairment in reasoning as the basis for the instability of choice, then that should be cited as an independent consideration.

## Practical implications

This optimised typology leads to several practical recommendations.

First, the capacity evaluators (judges, clinicians and others) should explicitly justify their determinations by reference to each specific MCA criteria. Our typology of rationales provides a kind of vocabulary for describing various impairment phenomena that can directly be linked to these MCA criteria.

Second, when the criterion of understanding is used, it should be used in its narrow sense (understanding-1 above) of grasping relevant information or concepts. It should not be used in one of the broader senses. The MCA does not explicitly prohibit broader uses or requires this narrow use of the understanding criterion. Our recommendation is only that, within what is permitted by the MCA, such a narrow use of understanding would create more transparent and reliable judgements and communications.

Third, the above two recommendations mean that the use or weigh criterion will encompass a large conceptual space. Of special note, the use or weigh criterion is shown to be the only MCA criterion with more than one capacity rationale falling under it. Thus, our recommendation is that whenever it is applied, evaluators make an effort to specify their rationale(s). This will help address a long-recognised problem of the breadth of the use or weigh criterion.^19–22^ As noted above, there is nothing inherently problematic about a mental capacity criterion that covers a lot of conceptual ground since decision making may involve complex abilities or concepts. We have written elsewhere^23^ that the space between comprehending (and retaining) relevant information and expressing a choice will likely involve multiple and varied abilities relevant to mental capacity; it just so happens that MCA covers this space with the broad criterion of ‘use or weigh.’

What we gain with the optimised typology is clarity of boundaries with important practical advantages. It makes judgements based on the use or weigh criterion more transparent and more accountable—and thereby, it is hoped, more reliable and valid. It could lead to more targeted efforts at supported decision making, especially if certain rationales are more prominently engaged with specific brain or psychiatric disorders.^18^ For example, a person with a learning disability might comprehend that a new housing option is being proposed, but have trouble imagining that information (rationale 2) such as what it would be like to live in that living situation; that person may especially benefit from actual visits or even a trial run in such accommodations, to support their decision-making. Finally, without drawing such boundaries consisting of discrete rationales, it is difficult to see how one could develop an instrument to measure the MCA abilities. With an optimised typology, it is at least possible.

## Objections

1. ***Given that the MCA criteria are legal criteria while the rationales are not explicitly endorsed in the MCA, why should the typology-based recommendations have any normative legal force?***

Unless the rationales are tied to and given as a justification for why P meets or fails one of the MCA criteria, then we agree they cannot ground a legally determinative conclusion. But it is difficult to see how an application of MCA criteria can be justified without resorting to the kind of rationales in the typology. A judge could render a judgement without an explanation—not giving any rationale at all—but that hardly seems ideal even if the judge had the legal authority to do so.^24^ Especially for a complex criterion such as ‘to use or weigh,’ how will we know that another evaluator or judge would have arrived at the same conclusion; or whether the judge’s application of that criterion was cogent?

Our recommendations are constrained by the actual judgements from the courts. It could turn out that judges might in the future reject one of the rationales that we identified. But to the extent that courts’ practices provide guidance, the typology simply organises and streamlines the set of rationales so that the MCA criteria can be applied more transparently and reliably. The typology can evolve as the law evolves.

1. ***The rationales themselves seem like ‘imperfect’ folk constructs that may not rigorously correspond to constructs related to decision making established by cognitive neuroscience.***

We agree that legal categories that engage scientific categories (impairments and disturbances that have fields of science attached to them) but are too divorced from the scientific evidence-base have less credibility as normative guides. But we do not think our typology poses any new or special issues in this regard. Indeed, to the extent that the rationales ought to be scientifically informed, not having a typology is a greater hindrance to that goal. Having a typology helps us see that some disorders or impairments of the mind or brain may make P more likely to exhibit decisional impairments tied to some rationales more than others^18^ and some rationales may cross disorders, suggesting caution with a narrowly diagnostic approach. Future work, informed by cognitive neuroscientific concepts, could improve our understanding of the constructs underlying the rationales and future science, informed by the rationales, could generate new scientific constructs.^25^ Of special relevance is the possibility that such advances can better pinpoint ways to support vulnerable persons with certain types of disorders.

1. ***The introduction of the appreciation rationale and the recommendation of using a narrow definition of understanding seem to reduce the MCA to the four abilities model which originated in the USA.*** 26 ***; this inappropriately imports a model not intended for England and Wales.***

First, that there is considerable conceptual overlap between what ‘functional capacity’ means in the four abilities model and in the MCA model should not be surprising. Indeed, too large a divergence between jurisdictions would raise concerns that the very idea of decision-making capacity is merely an arbitrary social construct.

Second, there is no good evidence that, taken as a whole, the English MCA policy process sought to exclude the relevance of the appreciation construct.^ix^ As can be seen from our typology, when the courts use the term, they are referring to the ability to apply the relevant information to one’s own situation; it thus refers to the ability to—in addition to grasping the relevant information—use that information as it applies to oneself. This requirement was endorsed by the Law Commission^27^ as it accepted that capacity requires something beyond mere understanding by citing situations where, for example, ‘certain… conditions cause people who are quite able to absorb information to arrive, inevitably, at decisions which are unconnected to the information or their understanding of it.’[at 3.17] In its discussion of what is required beyond mere absorption of information, it approvingly (twice) cites Thorpe J: ‘As Thorpe J said in *Re* C, some people can understand information but are prevented by their disability from being able to believe it.’^x^ The government’s subsequent Green Paper Who Decides^28^ largely followed the Law Commission’s recommendations on the definition of capacity, by making a distinction between the ability to understand relevant information and the ability of ‘using that information’ which ability could be stymied by ‘delusions or compulsions’ as when a person with schizophrenia ‘cannot believe what this doctors or financial advisors tell him…’^xi^[at 3.13]

Our typology shows that the courts make frequent use of the appreciation concept—combined as one category, appreciation accounted for 41% of all rationale texts in the documents we examined. This does not mean they used the actual term ‘appreciation’ (although they often did) in each case but rather that the concept was used (as in, eg, ‘understand that X applies to her’).

Third, rather than reducing the MCA into the four abilities model, the typology in fact draws out how the two models differ on a longstanding issue. The differences are instructive. Many writers have argued that influential models of decision-making capacity such as the four abilities model are ‘too cognitive’ and have difficulty handling cases where the apparent incapacity shown by P is due to non-cognitive factors having to do with dysfunctions in emotions or in valuing.^23 30–34^ There are disputes about whether the four abilities model’s appreciation criterion may^35^ or may not^33^ be able to cover such cases. But a recent systematic review on this topic^30^ shows that the role of emotions and values in capacity may be so complex that a single operational definition may be difficult. The typology of rationales for MCA criteria proposed here—which could continue to evolve—may be in a position to capture this complexity better. If there are indeed cases where a person’s incapacity is best accounted for by dysfunction in emotions or valuing, such incapacity could manifest not only as a problem in the ability to appreciate but also potentially as a problem with the ability to value or care, to reason (rigid inflexibility in face of evidence), or to think through decisions non-impulsively. This potential flexibility of the MCA framework with its ‘use or weigh’ criterion is simply the other side of the coin of the use or weigh criterion being very broad, and comes at the expense of requiring capacity evaluators to be familiar with multiple uses of a single concept.

## Conclusion

The recommendations based on the typology of rationales we derived from court judgements are consistent with the MCA. They provide a more concrete conceptual guidance than is available currently in applying the MCA criteria.

Importantly, in the long run, the typology could provide a framework for future research in several domains: legal research in further refining the typology’s relationship with future court judgements of capacity; psychometric research in measuring the MCA abilities in more systematic ways, as it will now be possible to construct an instrument to measure them, based on a typology that is grounded in the actual practice of the courts; and finally clinical and neuroscience research focusing on the relationship between the ethico-legal and scientific constructs of decision-making.

The guidance for capacity evaluators amounts to three relatively simple points: being explicit about which MCA criterion is being applied; using the criterion of understanding only in its narrow sense of grasping relevant information; and when applying the use or weigh criterion, being explicit about which rationale(s) are at play. These are simple recommendations but following them could considerably improve the practice of capacity assessments. The resulting evaluations will be more transparent, increasing reliability and accountability. It could lead to more targeted decision supports as well.

## Data Availability

No data are available. This paper does not report any original data.

## Footnotes

And the rulings from the associated appellate courts.

The reviewers suggested that our argument could support a revision of the MCA. Although we agree that our analysis shows some shortcomings of the current law, our aim here is more practical, to suggest ways to optimise its implementation to support those who are currently working under the law.

By ‘clinically grounded’ we mean only that clinical considerations are essential in explaining the causal role of “an impairment of, or a disturbance in the functioning of, the mind or the brain” in the determination of mental incapacity.[s.2 (1) MCA 2005] Others who have examined the CoP judgments with a more critical stance have raised concerns about the role of clinical experts’ influence on such judgments^15 16^

The Law Commission seems to have intended the narrow conception of understanding: “We explained…that certain compulsive conditions cause people who are quite able to *absorb* information to arrive, inevitably, at decisions which are unconnected to the information or their understanding of it.”[our emphasis; at 3.17] Indeed, one can read the Commission’s discussion of the criteria for capacity as largely reserving the use of ‘understanding’ to its narrow sense, and then explaining what other abilities in addition to that narrow conception of understanding are relevant for decision-making.

Again, we are comparing a specific narrow sense (understanding-1) to *several* broad uses (understanding-2). Indeed, sometimes ‘understanding’ even stands in as a synonym for being capable of making a decision, as when we say, “Do you think he *really* understands what’s going on?” as a way of conveying one’s general sense about a person’s capacity to make the decision in question.

The concept of ‘appreciation’ in the history of MCA has a long and complicated story, and we address its relevance to our typology in the section addressing objections below.

This is in keeping with the idea of restricting MCA ‘understanding’ criterion to mere comprehension only, rather than extending it to broader, related uses of ‘understanding’ that have to do with doing something with that which is comprehended. Otherwise, to admit one broad use of ‘understanding’ but not another broad use of it seems arbitrary.

The ‘communication’ criterion within the MCA is one that has been used in a wider fashion than had been anticipated by the Law Commission, which had expressly reserved it for either those who were unconscious or who retained cognitive functioning but were *completely* unable to communicate (para 3.13 of the 1995 Report). We did not encounter any published CoP cases where the focus was on determining the capacity of a person who is *completely* unable to communicate. That such cases do not make it to court is not surprising. However, the courts do encounter (although rarely), and extend the criterion of communication to, cases where the person cannot provide a stable or consistent choice.

It is true that in Northern Ireland, a jurisdiction closely associated with England and Wales, appreciation is explicitly included as a criterion,^6^ but especially in light of our analysis, an equivalent to which had not been carried out before this legislation was passed, we suggest that this can be seen as legislation ‘for the avoidance of doubt’ rather than a deliberate expansion of the criteria.

It is important to note that the belief aspect of the appreciation construct leaves room for scepticism, protest, subcultural belief or religious belief. Rather, it applies to instances of inability to believe relevant information due to impairment of mind or brain. For an extended discussion on how to apply the appreciation standard, see Kim 2010 at pp. 88–91.^8^

To be fair, at one point, the Law Commission seems to dismiss the need for the appreciation criterion by stating that their proposed definition of capacity does not have to deal with “the complications of asking whether a person needs to ‘appreciate’ information as well as understand it.”^27^ [at 3.17] But the Commission is referring to a broader conception of ‘appreciation’ which includes the notion of ‘emotional competence.’^29^
